# Trait mindfulness is protective for development of psychological distress in women with early breast cancer

**DOI:** 10.1080/21642850.2025.2517599

**Published:** 2025-06-17

**Authors:** Isabel Manica, Sílvia Almeida, Raquel Lemos, Berta Sousa, Albino J. Oliveira-Maia

**Affiliations:** a Champalimaud Research and Clinical Centre, Champalimaud Foundation, Lisbon, Portugal; b Faculdade de Ciências da Saúde e Enfermagem, Universidade Católica Portuguesa, Lisbon, Portugal; c Clinical and Health Psychology, Faculdade de Psicologia da Universidade de Lisboa, Lisbon, Portugal; d ISPA-Instituto Universitário de Cie^ncias Psicológicas, Sociais e da Vida, Lisbon, Portugal; e Breast Unit, Champalimaud Clinical Centre, Champalimaud Foundation, Lisbon, Portugal; f Clinical and Health Services Research, Faculty of Medicine of the University of Porto (FMUP), Porto, Portugal; g NOVA Medical School (NMS), Faculdade de Cie^ncias Médicas (FCM), Universidade NOVA de Lisboa, Lisbon, Portugal

**Keywords:** Anxiety, breast cancer, depression, mindfulness, somatic symptoms

## Abstract

**Background:**

Breast cancer causes significant psychological and physical burden, with survivors often reporting persistent psychological symptoms, such as anxiety and depression, along with somatic symptoms like fatigue and pain. Psychological factors may protect from the development of long-term distress and help identify patients with greater needs for supervision and/or care. Here we aimed to study the predictive role of mindfulness as a trait in determining affective and somatic symptoms 12 months after cancer diagnosis.

**Methods:**

Women with a diagnosis of breast cancer were recruited at the onset of systemic treatments and compared with healthy women from the general population. Over a 12-month period, participants were periodically assessed using the Mindfulness Attention Awareness Scale (MAAS), the Hospital Anxiety and Depression Scale, the European Organization for Research and Treatment of Cancer Quality of Life Questionnaire – Core 30 and the Positive and Negative Affect Scale. Regression models were employed to assess the predictive associations of baseline mindfulness trait with symptoms after 12 months.

**Results:**

The study included 282 participants, 243 of whom contributed complete data for analysis. The Portuguese version of MAAS demonstrated adequate psychometric properties for patients with breast cancer, supporting its use to address our main aim. Mindfulness trait scores remained stable across time, did not differ significantly between patients and healthy participants, and were similarly stable across time for patients undergoing different treatments. Also in the clinical group, MAAS scores at baseline significantly predicted affective, but not somatic symptoms, 12 months later, with higher levels of mindfulness predicting more clinically significant distress.

**Conclusions:**

In women with a recent diagnosis of breast cancer, mindfulness trait appears unaffected by diagnosis or its treatments, serving as a protective factor against affective, but not somatic, symptoms, in the first year following diagnosis.

## Background

Breast cancer (BC) has increased in prevalence, with numbers exceeding those of lung cancer, becoming the most common subtype of cancer worldwide (Sung et al., 2021). Importantly, the number of BC survivors has also increased, raising concerns regarding long-term quality of life (DeSantis et al., 2019). Diagnosis and treatment of cancer can have a significant impact on physical and psychological well-being, and thus on quality of life (Sarenmalm et al., 2014). Patients often experience stress, symptoms of depression and anxiety, as well as somatic symptoms, such as pain and fatigue, that may persist and lead to long-term complications (Antoni & Dhabhar, 2019; Singer et al., 2010). In addition to emotional distress and general somatic symptoms, BC often disrupts patients’ relationship with their bodies, leading to disturbances in body image, alterations in sexual functioning, and a sense of disconnection from the physical self. Treatments such as mastectomy, chemotherapy, and hormone therapy can result in visible and physical changes – such as scarring, hair loss, and lymphedema – that may undermine perceived femininity, self-esteem, and intimacy (Sebri et al., 2022; Sebri & Pravettoni, 2023). These bodily transformations are closely linked to identity and emotional regulation, potentially exacerbating psychological distress (Sebri et al., 2022). Several therapeutic approaches have been used to reduce the burden of cancer diagnosis and treatment, aiming to improve overall well-being. These include pharmacological treatments (Holland & Rowland, 1989; Ruano et al., 2022), as well as psychosocial interventions (Blanco et al., 2019; Guarino et al., 2020; Watson, 1983; Ye et al., 2018), including mindfulness-based interventions (Haller et al., 2017; Li et al., 2023; McCloy et al., 2022; Zhang et al., 2019).

Mindfulness is defined as a mental state where one focuses attention on present experiences, thoughts, and emotions, without judgment (Kabat-Zinn et al., 1992). It involves intentionally bringing awareness to the current moment, without considering the past or future (Brown & Ryan, 2003). It can be considered as a trait/disposition or a state, with ‘trait mindfulness’ denoting an inherent tendency to sustain a state of awareness towards the present moment in any given time (Medvedev et al., 2017; Witek-Janusek et al., 2008), and ‘state mindfulness’ being the non-judgmental and present-centered awareness that one experiences in a particular moment (Medvedev et al., 2017). The practice of mindfulness has been linked to psychological health (Polizzi et al., 2018; Tomlinson et al., 2018), including negative associations with affective symptoms and positive correlations with health and well-being (Alsubaie et al., 2017; Carpenter et al., 2019). Indeed, mindfulness-based interventions have been proposed to be effective in reducing symptoms of anxiety and depression in patients and survivors of BC (Chayadi et al., 2022; Hilton et al., 2017; Piet et al., 2012). Furthermore, a randomized controlled trial of a 8-week mindfulness-based stress reduction (MBSR) program described a significant reduction of anxiety and improved quality of life among BC survivors (Sakki et al., 2022), similarly to what has been found for depression and anxiety in the general population (Hofmann et al., 2010; Khoury et al., 2015). Mindfulness-based interventions have been shown to reduce physical burden and somatic symptoms – such as pain, fatigue, and sleep disturbance – in patients with various cancers (Garland et al., 2014; Johns et al., 2015; Lengacher et al., 2016), though other studies report no such effects (Khoury et al., 2015; Ledesma & Kumano, 2009).

While the benefits of mindfulness-based interventions are well documented (Johns et al., 2015; Lengacher et al., 2016), the potential protective role of trait mindfulness in the development of psychological symptoms remains less explored, particularly in patients with cancer. A protective role for dispositional mindfulness has been reported in other populations, with the level of trait mindfulness related with the severity of the psychological sequelae in individuals with adverse childhood experiences (Daigneault et al., 2016). In high school students exposed to high levels of daily stress, low levels of mindfulness were also associated with more symptoms of depression and anxiety (Cortazar & Calvete, 2019; Marks et al., 2010), with other studies also describing negative correlations between trait mindfulness and symptoms of depression, anxiety and eating disorders (Lattimore, 2020; Tomlinson et al., 2018). However, in these populations, the dissociation between the potential effects of stressors on mindfulness and on psychopathology is frequently difficult to disentangle.

Given the protective effects of trait mindfulness in other populations, and the well-established benefits of mindfulness-based interventions for reduction of distress in patients with cancer, the question arises as to whether trait mindfulness may also play a protective role in this patient population, particularly with regards to psychological and somatic symptoms. To address these questions, here we investigated whether trait mindfulness, assessed early in the cancer trajectory, predicts affective and somatic symptoms one-year after diagnosis. We hypothesized that higher baseline mindfulness would be associated with lower anxiety, depression, pain, and fatigue 12 months later.

## Participants

Women with early breast cancer – stages I–III – were recruited as part of a multi-centre clinical pilot study *BOUNCE* (Predicting Effective Adaptation to Breast Cancer to Help Women to BOUNCE back – H2020 Project number 777167; Pettini et al., 2022). The data for the present study was collected at the Breast and Neuropsychiatry Units of the Champalimaud Clinical Centre, the centre in Lisbon, Portugal. Patients completed a series of questionnaires including measures of resilience, quality of life, coping, mood, sociodemographic and clinical characteristics, every 3 months for 18 months (Pettini et al., 2022). For this study, only two assessments were considered: at baseline, prior to the start of systemic treatments, and 12 months later. The eligibility criteria included: female patients with 18–70 years of age at the time of the enrollment in the study, confirmed diagnosis of invasive BC ranging from tumor stage I–III, scheduled for local treatment with or without adjuvant radiotherapy and any type of systemic treatment (Pettini et al., 2022). A control group without history of BC was recruited from the general population through social media announcements and flyers. Selection of participants for the control group followed a demographic frequency matching strategy, to promote a similar distribution of age and years of education relative to patients. Participants from the clinical group were excluded in the presence of distant metastases, if they had a history of another malignancy or contralateral invasive BC within the last five years (with exception of cured basal cell carcinoma of skin or carcinoma in situ of the uterine cervix), history of early-onset (i.e. before 40 years of age) mental disorder (i.e. schizophrenia, psychosis, bipolar disorder, diagnosis of major depression), severe neurologic disorder (i.e. a neurodegenerative disorder, dementia), major surgery for severe disease or trauma which could affect the psychosocial wellbeing (for example, major heart or abdominal surgery) within 4 weeks prior to study entry, or lack of complete recovery from the effects of such surgery, treatment for any major illness in the last half-year, pregnancy or breastfeeding at the time of recruitment, non-fluency in Portuguese or basic education outside of Portugal. In addition to the previous criteria, that were also applied to healthy volunteers whenever appropriate, participants from either group were also excluded if they presented other diagnosed concomitant diseases such as clinically significant (i.e. active) cardiac disease (e.g. congestive heart failure, symptomatic coronary artery disease, or cardiac arrhythmia not well controlled with medication) or myocardial infarction within the last 12 months.

## Measures

### Sociodemographic, lifestyle questionnaire and medical data

A questionnaire was developed for the BOUNCE study to assess sociodemographic and lifestyle variables, including age, education, marital and employment status (Pettini et al., 2022).

### Mindful attention and awareness scale (MAAS)

The MAAS is a 15-item scale that was developed to measure the level of trait mindfulness in people without meditation experience (Brown & Ryan, 2003). The scale ranges on a 6-point Likert Scale, ranging from 1 (‘almost always’) to 6 (‘almost never’). MAAS total score represents the average of all the items and can range from 1 to 6, with higher scores indicating higher levels of trait mindfulness. The MAAS has been widely used in research and has been shown to be a valid and reliable measure of trait mindfulness (Catak, 2012; de Bruin et al., 2011), including in patients with cancer (Carlson & Brown, 2005; Nooripour et al., 2022), and in a non-cancer Portuguese population (Gregório & Pinto-Gouveia, 2013). The validation paper for the Portuguese population showed a clear unidimensional structure and a high Cronbach’s alpha (0.9). Those authors suggested that item 13 should be removed, due to increased alpha if item removed, as well as lowest loading factor and R^2^ (Gregório & Pinto-Gouveia, 2013). For the purpose of the present study, this item was considered. Since MAAS has not been validated for a population of Portuguese patients with cancer, we started by studying its psychometric properties, following the same approach as described in our previous work (Almeida et al., 2023; Lemos et al., 2022).

### European organization for research and treatment of cancer quality of life questionnaire – core 30 (EORTC-QLQ-C30)

The EORTC-QLQ-C30 is a quality-of-life questionnaire developed by the European Organization for the Research and Treatment of Cancer (Aaronson et al., 1993). It is composed of 30 items: 24 are organized in a multi-item scale and the remaining 6 refer to isolated symptoms that intend to reflect the multidimensionality of quality of life. This questionnaire it is organized in five functional subscales (social, emotional, cognitive, physical and role), a global health/QoL scale, three symptoms’ subscales (e.g.: fatigue, pain) and single items related with additional symptoms (e.g.: dyspnea). The score for each subscale ranges from 0 to 100%, with higher values reflecting better quality of life, except for symptom subscales where higher values represent greater symptom intensity. In the Portuguese validation, performed in patients with cancer, the scale presented high internal consistency: 0.89 for the total scale, 0.75–0.91 for the functional scales; and 0.74–0.87 for the symptom scales (Pais-Ribeiro et al., 2008).

### Positive and negative affect scale (PANAS) – short form

The PANAS short form is a widely recognized and validated assessment tool used to measure both positive and negative emotional states experienced by individuals (Thompson, 2007). The PANAS short form consists of two distinct subscales: Positive Affect (PA) and Negative Affect (NA). The PA subscale is designed to capture a person's positive emotional experiences and includes items related to feelings of enthusiasm, inspiration, determination, attentiveness, and activity. Conversely, the NA subscale focuses on negative emotional states and encompasses items assessing feelings of stress, fear, nervousness, guilt and scared. Participants rate the extent to which they experience each of these emotions on a Likert scale ranging from 1 (indicating ‘very slightly or not at all’) to 5 (representing ‘extremely’). Total score for each subscale ranges from 5 to 25. The Portuguese short version of this scale demonstrated good psychometric properties (Costa Galinha et al., 2014).

### Hospital anxiety and depression scale (HADS)

The Hospital Anxiety and Depression Scale (Zigmond & Snaith, 1983) is a self-reported questionnaire used to screen for symptoms of depression and anxiety. It consists of 14 items: seven assess anxiety and the other seven assess depression. The scale uses a 4-point Likert-Scale, ranging from 0 to 3. For both subscales, higher scores are indicative of greater psychological distress. In the validation study with the Portuguese population, HADS presented a good internal consistency for both subscales (0.71 for anxiety and 0.81 for depression scale) (Pais-Ribeiro et al., 2007).

## Procedures

The Ethics Committee of Champalimaud Foundation reviewed and approved the study methods and protocols (*approval number: 2018052102*). Participants were considered only after providing written informed consent, and the study was carried out following the Declaration of Helsinki. The data was handled in compliance with international, EU, and national laws, including the EU General Data Protection Regulation.

## Data analysis

JASP version 0.16.4., an open-source statistical analysis software built on R-package lavaan, was used to perform the statistical analysis. The use of parametric statistics was supported by the large sample size, in accordance with the central limit theorem. Descriptive statistics were employed for sociodemographic, clinical, and psychometric data, including means and standard deviations (SD) and percentages. Comparisons were performed by t-test for independent samples for continuous variables, or chi-square test for ordinal variables.

Since there are no validated scales to assess trait mindfulness in Portuguese patients with cancer, we started by exploring the psychometric properties of the MAAS in this population. To assess dimensionality, a Confirmatory Factor Analysis (CFA) was performed, where a model with one factor structure was specified according to the original proposed structure. The model's fit was evaluated using several indices, including non-significant chi-square test, the comparative fit index (CFI) and the Tucker-Lewis index (TLI). A CFI value of 0.90 or higher and a TLI value of 0.95 or higher indicated good or very good model fit. Additionally, the Root Mean Square Error of Approximation (RMSEA) was used, with values of 0.08 or lower and 0.05 or lower used to indicate acceptable or very good fit, respectively (Schermelleh-Engel et al., 2003). The analysis also included an examination of factor loadings (λ) which show the strength of the relationship between the latent variables and the observed variables. Factor loadings with a score of 0.40 or higher were a good indicator of item quality (Gana & Broc, 2019). Reliability of the MAAS scale was assessed using Cronbach's alpha (α) and McDonald’s Omega (ω), with values of 0.7 or higher indicating good internal consistency (Hair, 2011). The construct validity of MAAS was assessed in the clinical sample using Person’s Correlation Coefficients, with the emotional functional score of the EORTC-QLQ-C30 for convergent validity and PANAS NA subscale for divergent validity. To compare the MAAS scores between different treatment groups across assessment time points, a mixed-effects ANOVA was fitted in GraphPad Prism 8.0.1. This method accounted for missing data using the Geisser-Greenhouse correction.

We conducted five hierarchical linear regression models to assess how well the MAAS score at baseline predicts affective and somatic symptoms using the Enter Method. We used the MAAS score as the independent variable and considered fatigue, pain (as measured by EORTC QLQ-C30), depression, anxiety, and psychological distress (as measured by HADS) at 12 months as the dependent variable in separate models. In each model, sociodemographic and clinical variables (age, education level, marital status, employment status, and primary cancer treatment), were entered in the first step, followed by the MAAS score entered in the second step. Categorical variables were transformed into binary variables to facilitate the analysis: educational level was coded as 0 for high school or less and 1 for university-level education; marital status was coded as 0 for living with a partner and 1 for living alone; employment status was coded as 0 for unemployed/retired and 1 for employed; cancer treatment was coded as 0 for chemotherapy and 1 for endocrine therapy. This approach allowed us to consider the impact of these factors on the relationships under investigation while accounting for potential confounds.

Additionally, a logistic regression with Forward Method was performed to further explore whether MAAS scores at baseline predicted reaching a diagnostic threshold on the Hospital Anxiety and Depression Scale (HADS) at 12 months. HADS total score was dichotomized according to diagnosis cut offs (≥ 13 for Total Scale and ≥ 8 for Anxiety and Depression Subscales), as recommended in the available literature (Wu et al., 2021; Snijkers et al., 2021; Singer et al., 2009). MAAS discriminant predictive power was also verified by analyzing sensibility and specificity of the scale and calculating the Area under the curve (AUC) (Mandrekar, 2010). The AUC provides a measure of the model's ability to correctly classify individuals with and without clinically relevant affective symptoms, with values closer to 1.0 indicating better discriminative performance. In addition, we computed sensitivity and specificity to evaluate the model’s ability to correctly identify patients who do and do not meet diagnostic criteria.

In all analyses, results with an alpha-level (p) < 0.05 were considered statistically significant.

## Results

### Descriptive statistics

Our sample included 189 women with early, non-metastatic breast cancer, 160 of whom completed all the questionnaires used for this study (Table S1). Among these, first systemic treatment was either chemotherapy (n = 90) or endocrine therapy (n = 70). Data on sociodemographic and clinical characteristics are displayed in Table 1. The mean age of the sample is 51.2 years (SD = 8.9). The majority of the subjects had a university degree (bachelor, master or doctoral: 73.8%), 74.4% were married and 85% were employed. A sample of 93 women, frequency-matched according to age and education, was also included, and 83 filled-in all the questionnaires. The mean age of this sample was 49.9 (SD = 8.8). Similarly to the clinical sample, the majority of healthy volunteers had a university degree (71.3%), 64.4% were married and 88.5% were employed. As expected, no differences between healthy and clinical samples were found in any sociodemographic variable.

**Table 1.** Sociodemographic characteristics and difference between both samples

	Clinical Sample (N = 160)		Healthy Sample (N = 83)			
	n	%	%	n	t	*p*
Age, mean (SD)	51.2	8.9	49.9	8.8	**1**.**1**	**0**.**3**
					**χ2**	***p***
Age Group						
≤ 40 years	15	9.4	13	14.9	**2**.**7**	**0**.**4**
41–50 years	69	43.1	36	41.4		
51–60 years	49	30.6	28	32.2		
> 60 years	27	16.9	10	11.5		
Education Level						
Non-University	42	26.3	25	28.7	**0**.**18**	**0**.**6**
Graduate degree	118	73.8	62	71.3		
Marital Status						
Single/Engaged	21	13.1	16	18.4	**2**.**7**	**0**.**3**
Married	119	74.4	56	64.4		
Divorced/widowed	20	12.5	15	17.2		
Employment Status						
Employed	136	85.0	77	88.5	**0**.**8**	**0**.**7**
No Formal Employment	10	6.3	5	5.8		
Retired	14	8.8	5	5.8		

### Psychometric properties of MAAS

**Figure 1.**
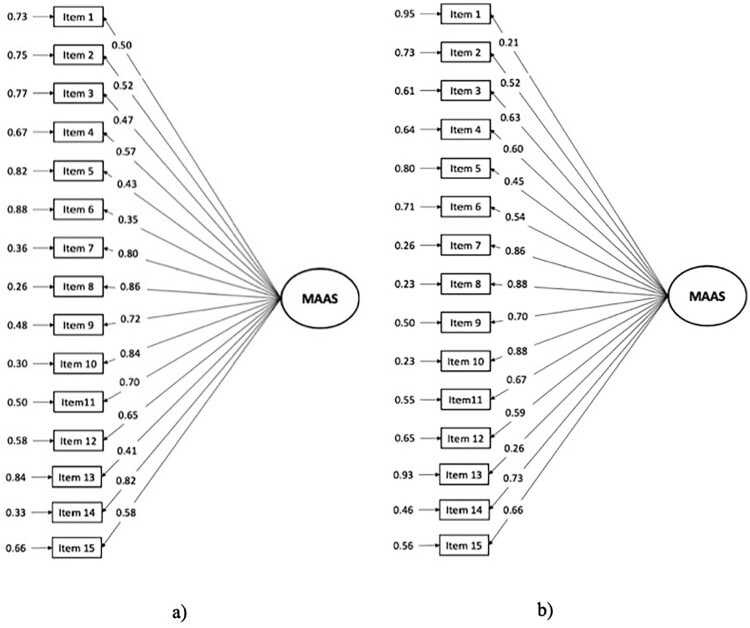
The figure shows standardized parameter estimates (on the left) and measurement errors (on the right) for the 15 items of the MAAS in a one factor CFA conducted either among a sample of Portuguese women with breast cancer (panel a) or a sample of Portuguese women without breast cancer (panel b).

A CFA was performed to confirm the single factor structure of the Portuguese version of MAAS in patients with breast cancer, as described previously for a non-clinical population (Gregório & Pinto-Gouveia, 2013). Goodness-of-fit indices of the general model demonstrated good values, with an adequate fit to the data of the clinical sample, confirming a clear single factor structure: χ^2^ = 161.59, *p* < .001; χ^2^/df = 1.79; CFI = 0.98; TLI = 0.98; RMSEA = 0.07 (Figure 1a). Globally, most items presented good adjustment, except item 6 (‘I forget a person’s name almost as soon as I’ve been told it for the first time’) that presented a loading factor marginally under the recommended minimum value of 0.4 (λ = 0.35). This item was conserved since the model fit to the data did not improve significantly when it was removed (data not shown). As mentioned above, in the original validation study for the Portuguese population (Gregório & Pinto-Gouveia, 2013), removal of item 13 was proposed since it presented the lowest loading factor. While loading of this factor was appropriate in our sample (λ = 0.41), we nevertheless performed an additional CFA excluding item 13, and found that the goodness-of-fit values for this model were very similar to the first model: χ^2^ = 110.8, *p* < .001; χ^2^/df = 1.44; CFI = 0.99; TLI = 0.99; RMSEA = 0.05. However, in a CFA of data from the healthy volunteer sample, while overall fit of the model was adequate (χ^2^ = 104.6, *p* = .14; χ^2^/df = 1.16; CFI = 0.99; TLI = 0.99; RMSEA = 0.04; Figure 1b), item 13 (‘ I find myself preoccupied with the future or the past’), as well as item 1 (‘I could be experiencing some emotion and not be conscious of it until sometime later’) presented loading factor indices below the recommend value of 0.4 (λ = 0.26 and λ = 0.21 respectively).

For the BC sample, the MAAS showed excellent reliability (ω = 0.87, 90% CI: 0.89–0.92; α = 0.87, 90% CI: 0.88–0.92). The values remained stable with removal of any item (ω: 0.85–0.87; α: 0.85–0.87), and corrected item-total correlations ranged between 0.65 and 0.99 (Table 2). In the healthy volunteer sample, the scale also showed excellent reliability (ω = 0.87, 90% CI: 0.89–0.92; α = 0.88, 90% CI: 0.88–0.92), the values remained stable with removal of any item (ω: 0.86–0.88; α: 0.86–0.88) and corrected item-total correlations ranged between 0.64 and 0.97 (Table S2).

**Table 2.** Table item summaries and reliability parameters for the clinical sample.

Item	Statistics			Percentage of Endorsement							Reliability		
	M (SD)	Sk	Ku	1	2	3	4	5	6	Total	α if item deleted	ω if item deleted	Item-Total Correlation
1	4.62 (1.3)	−0.64	−0.53	0.6	6.8	13.1	20	27.5	31.9	100	0.86	0.86	0.77
2	4.95 (1.2)	−1.01	0.17	0.6	4.3	8.8	17.5	22.5	46.3	100	0.86	0.86	0.86
3	4.41 (1.3)	−0.49	−0.63	0.6	8.8	14.4	23.1	30.6	22.5	100	0.86	0.86	0.99
4	4.06 (1.5)	−0.37	−0.91	5.6	12.5	18.1	18.1	25.6	20	100	0.86	0.86	0.99
5	3.93 (1.5)	−0.15	−1.09	4.4	15	23.8	15	23.8	18.1	100	0.87	0.87	0.99
6	3.63 (1.8)	0.03	−1.4	12.5	20.6	17.5	12.5	13.8	23.1	100	0.88	0.87	0.99
7	4.33 (1.3)	−0.39	−0.68	1.3	7.5	18.8	23.1	28.1	21.3	100	0.85	0.85	0.86
8	4.73 (1.1)	−0.57	−0.45	0	3.1	11.3	23.8	32.5	29.4	100	0.86	0.86	0.86
9	4.62 (1.1)	−0,50	−0.30	0.6	1.9	15	24.4	33,75	24.4	100	0.86	0.85	0.99
10	4.58 (1.1)	−0.45	−0.60	0	3.8	15	23.8	35	22.5	100	0.86	0.86	0.99
11	4.12 (1.3)	−0.19	−0.98	0.6	11.3	23.1	20.6	29.4	15	100	0.86	0.85	0.86
12	4.58 (1.3)	−0.60	−0.71	0.6	7.5	15.6	16.8	28.1	31.3	100	0.86	0.86	0.65
13	3.48 (1.3)	0.23	−0.68	5	18.1	33.8	18.1	16.9	8.1	100	0.87	0.87	0.86
14	4.42 (1.1)	−0.43	−0.54	0	6.3	15	26.3	35.6	16.9	100	0.86	0.85	0.86
15	5.19 (1.2)	−1.34	0.91	0	3.8	6.9	11.3	23.1	55	100	0.86	0.86	0.99

For each item of the MAAS scale, the mean (M), standard deviation (SD) and the percentage of endorsement for each possible item score (range 1-6) is presented. Sk = Skewness; Ku = Kurtosis, α if item deleted = Cronbach’s α; ω if item deleted = McDonalds ω if item deleted.

Among women with breast cancer, MAAS showed good convergent validity with the emotional functional score of the EORTC QoL C30 (r = 0.2; *p* = 0.01) and good divergent validity with the PANAS NA score (r = −0.22, *p* = 0.001).

### MAAS scores in patients with breast cancer

**Figure 2.**
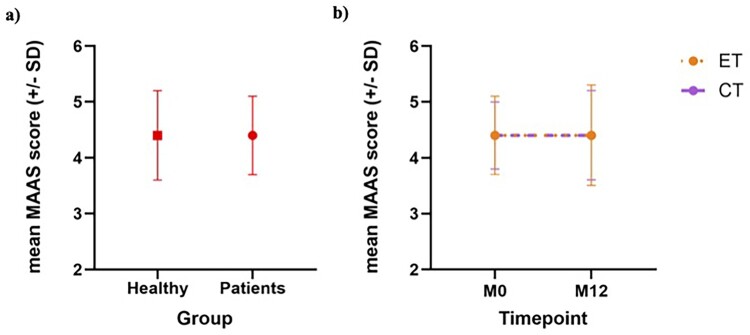
Mindfulness scores comparison in breast cancer patients and controls, and across treatment groups over time. The figure shows the MAAS mean scores and standard deviations (SD) for breast cancer patients and healthy controls at baseline (panel a) and for the chemotherapy (CT; in purple) and endocrine (ET; in orange) treatment groups at M0 (baseline) and M12 (12 months) (panel b). Error Bars: ±1SD

At baseline, we did not find significant differences in MAAS scores between the Healthy (4.4 ± 0.8) and Clinical (4.4 ± 0.7) samples (t(_245_) = −0.36, *p* = 0.71; Figure 2a). A mixed-effects ANOVA was used to examine the main effects of treatment group (chemotherapy vs. endocrine therapy) and time (baseline vs. 12 months) on MAAS scores. The results revealed that neither group (F (1, 158) = 0.07 *p* = 0.98) nor time (F(1, 122) = 0.22, *p* = 0.63) had significant effects on MAAS scores. The interaction between group and time was also not significant (F (1, 122) = 0.18, *p* = 0.66), further supporting that both groups had similar and stable levels of mindfulness across time (Figure 2b). A similar exploratory analysis of MAAS scores in this population according to age did not reveal any age-dependent effects (Figure S1).

### Mindfulness as a predictor of somatic and affective symptomatology

To investigate whether mindfulness can predict somatic and affective symptoms 12 months after cancer diagnosis (Figure S2), we conducted five hierarchical linear regressions using the Enter Method (Table 3). For each model, sociodemographic and clinical covariates were entered in the first step, and MAAS total score at baseline was entered in the second step. A first linear regression was performed using fatigue (as measured in the EORTC QLQ-C30) at 12 months as the dependent variable. In this model, step 1 explained 12% of variance (R^2^ = 0.12, adjusted R^2^ = 0.08), with only type of treatment having a significant effect on fatigue (β = −0.28, *p* = 0.003). Adding MAAS total score in Step 2 explained an additional 0.8% of variance (ΔR² = 0.008, *p* = 0.34), and MAAS at baseline was not a significant predictor of fatigue (β = −0.09, *p* = 0.34), with type of treatment preserving effects on fatigue (β = −0.28, *p* = 0.003). Similarly, for pain, as measured in the EORTC QLQ-C30 at 12 months, Step 1 explained 9% of pain symptoms (R^2^ = 0.09, adjusted R^2^ = 0.05) and Step 2 added only 0.5% of variance (ΔR² = 0.005, *p* = 0.43). MAAS total score at baseline was not a significant predictor of pain (β = −0.08, *p* = 0.43), while type of treatment was again a significant predictor (β = −0.24, *p* = 0.01). The other three linear regressions had HADS total score, HADS anxiety or HADS depression as dependent variables, and the models showed that adding MAAS total score in Step 2 significantly improved prediction. For distress (as measured by HADS total score), Step 1 explained 4% of the variance (R^2^ = 0.04, adjusted R^2 ^= −0.01), and Step 2 added 12% (ΔR² = 0.12, *p* < 0.001), with MAAS total score emerging as a significant predictor (β = −0.36, *p* < 0.001). For anxiety (as measured by HADS Anxiety), MAAS added 13% of variance (ΔR² = 0.13, *p* < 0.001), beyond the 5% explained by Step 1 (R^2^ = 0.05, adjusted R^2^ = 0.01), and was a significant predictor (β = −0.37, *p* < 0.001). For depression, Step 1 explained 3% of the variance (R^2^ = 0.03, adjusted R^2 ^= −0.02), and Step 2 added 8% (ΔR² = 0.08, *p* = 0.003), with MAAS total score again being a significant predictor (β = −0.29, *p* = 0.003). None of the other covariables were significant predictors of the affective symptoms.

**Table 3.** Hierarchical linear regressions: Prediction of somatic and affective symptoms at 12 months by MAAS total score at baseline.

	Step	R²	Adj. R²	ΔR²	ΔF (*p*)	MAAS β (*p*)	Other Sig. Covariables
Fatigue	1	0.12	0.08	–	–	–	Type of Treatment (β = −0.28, *p* = 0.003)
	2	0.13	0.08	0.008	0.93 (*p* = 0.34)	−0.09 (*p* = 0.34)	Type of Treatment (β = −0.28, *p* = 0.003)
Pain	1	0.09	0.05	–	–	–	Type of Treatment (β = −0.05, *p* = 0.01)
	2	0.09	0.04	0.005	0.62 (*p* = 0.43)	−0.08 (*p* = .0.43)	Type of Treatment (β = −0.24, *p* = 0.01)
Distress	1	0.04	−0.01	–	–	–	*None*
	2	0.16	0.11	0.12	15.47 (*p* < 0.001)	−0.36 (*p* < 0.001)	*None*
Anxiety	1	0.05	0.01	–	–	–	*None*
	2	0.19	0.14	0.13	17.35 (*p* < 0.001)	−0.37 (*p* < 0.001)	*None*
Depression	1	0.03	−0.02				*None*
	2	0.11	0.06	0.08	9.42 (*p* = 0.003)	−0.29 (*p* = 0.003)	*None*

Note: R^2^: Coefficient of determination (proportion of variance explained by the model); Adj. R^2^: Adjusted Coefficient of determination (corrected for the number of predictors in the model); ΔR²: Change in R^2^ from Step 1 to Step 2; ΔF (*p*) = F-test and *p*-value for the significance of the R² change between steps; MAAS β (*p*) = Standardized regression coefficient and *p*-value for the MAAS total score predictor.

To explore the previous finding in greater detail, namely if MAAS scores at baseline predicted the likelihood of having clinically relevant symptoms on the HADS at 12 months, logistic regression analyses using the Forward Method were performed. MAAS score at baseline was a significant predictor of the likelihood of having HADS total score above the threshold for clinical significance at 12 months (Β = −0.87, χ^2^_Wald_(1) = 8.84, *p* = 0.003, *OR *= 0.42), indicating that higher baseline mindfulness is associated with a lower likelihood of having clinically significant distress according to the HADS total score, 12 months after initial diagnosis of breast cancer. Equivalent analyses performed separately for each HADS subscales (Anxiety and Depression), revealed significant protection from development of anxiety (Β = −0.99, χ^2^_Wald_(1) = 10.4, *p* = 0.001, *OR *= 0.37), but only a borderline effect for depression (Β = −0.61, χ^2^_Wald_ (1) = 3.8, *p* = 0.05, *OR *= 0.55).

To ensure that the significance of the previous model represents prediction of distress rather than reflecting circular associations between distress and MAAS at both timepoints, we further tested MAAS for accuracy in predicting distress only among participants with scores below the HADS cut-off at baseline (n = 116). In this model, the likelihood of MAAS predicting clinically significant distress symptoms (Β = −0.5, χ^2^_Wald_ (1) = 48.2, *p* < 0.001, *OR *= 0.62) seemed similar, or maybe even improved, relative to that observed in the full sample, with the discriminatory power (AUC = 0.63, *p* = 0.01), sensitivity (83%; 95% CI [52%–98%]) and specificity (52%; 95% CI [40%–64%]) of MAAS further confirming an acceptable predictive power of distress by the MAAS.

## Discussion

This study aimed to examine whether a predisposition for mindfulness is associated with resilience to affective and somatic symptoms in patients with breast cancer. We assessed trait mindfulness using the MAAS in a sample of Portuguese women with BC and a matched group of healthy controls. We started by studying the psychometric properties of the scale and found that it is a valid and reliable measure to use in this population. Importantly, MAAS scores were similar between women with BC and education and age-matched healthy controls. In women with BC, MAAS score did not differ according to treatment types and was stable across time in the first year after diagnosis. Considering the validity and stability of MAAS and its apparent indifference to the diagnosis of cancer and to cancer treatments, supporting its role in assessment of a trait, we could address our main objective. We found that MAAS scores at baseline were predictive of protection from the development of clinically significant distress, but not of somatic symptoms, after 12 months.

The psychometric evaluation of the Portuguese version of the MAAS in women with BC supported a unidimensional factor structure with good fit, consistent with previous studies in both healthy individuals and patients with cancer (Brown & Ryan, 2003; Gregório & Pinto-Gouveia, 2013; MacKillop & Anderson, 2007; Nooripour et al., 2022). Although three items showed lower factor loadings (item 6 for patients with cancer and item 1 and 13 for healthy sample), their retention did not compromise the overall model fit and is consistent with earlier findings in Portuguese samples (Carlson & Brown, 2005) and population with cancer (Gregório & Pinto-Gouveia, 2013; Nooripour et al., 2022). The scale demonstrated very good internal consistency (α = 0.87). Convergent validity was confirmed by significant correlations between MAAS and the functional emotional subscale of EORTC QLQ-C30, indicating that higher levels of mindfulness were associated with better emotional functioning, as expected. Similarly, the correlation between MAAS and PANAS NA confirmed divergent validity, where higher levels of mindfulness were associated with lower negative affect.

We observed no significant differences in trait mindfulness, as measured by the MAAS scale, between healthy and clinical samples. Moreover, the mean MAAS scores obtained in this study were comparable to those reported previously for the Portuguese population (Gregório & Pinto-Gouveia, 2013), as well as in other cultures (Catak, 2012; de Bruin et al., 2011). Further support for the robust assessment of a trait-like characteristic by the MAAS was obtained among BC patients, for whom levels of dispositional mindfulness were similar between the chemotherapy and endocrine therapy groups and did not change significantly over the course of 12 months of treatment. This finding is consistent with previous studies that also did not find significant differences in mindfulness levels between BC patients undergoing different types of treatments (Witek-Janusek et al., 2008). Our results suggest that the lack of differences between the treatment groups may be due to the nature of the measure used, since the MAAS scale assesses dispositional mindfulness – a relatively stable trait – rather than a momentary and context-specific state of mind (Medvedev et al., 2017). In sum, these findings support using the MAAS to address our main aim.

We then investigated whether mindfulness at baseline could predict affective and somatic symptoms 12 months after the initial BC diagnosis. Our linear regression analyses revealed a relationship between mindfulness trait and affective symptoms (distress, anxiety and depression symptoms), but not somatic symptoms, namely pain and fatigue. Importantly, trait mindfulness was inversely associated with affective symptoms, suggesting a protective role against their development one year later. Furthermore, in logistic regression models, the odds of experiencing clinically significant distress at 12 months reduced significantly for each point increase of MAAS at baseline, even when excluding patients exhibiting significant distress at baseline. This finding adds to growing evidence that mindfulness may help predict and manage affective symptoms. Indeed, mindfulness-based interventions, such as Mindfulness-Based Stress Reduction and Mindfulness-Based Cognitive Therapy, have been shown to be effective in reducing symptoms of anxiety and depression (Hofmann et al., 2010; Khoury et al., 2015). These interventions typically involve training individuals in mindfulness meditation techniques and encouraging the development of mindful awareness in daily life (Baer, 2019).

It is important to note that this study did not find a significant relationship between mindfulness and somatic symptoms. This is consistent with the results of previous studies and meta-analyses that have found stronger and more consistent effects of mindfulness on depression and anxiety, compared to with physical outcomes such as pain and physical functioning (Dobos et al., 2015; Khoury et al., 2015). Research on the relationship between mindfulness and somatic symptoms in patients with cancer has produced mixed results. Some studies found a significant negative relationship between mindfulness and somatic symptoms, indicating that greater mindfulness is associated with lower levels of somatic symptoms such as pain, fatigue, and nausea (Garland et al., 2014; Johns et al., 2015; Lengacher et al., 2016), while others have not found such a relationship (Khoury et al., 2015; Ledesma & Kumano, 2009; Luberto et al., 2018). It is worth noting that somatic symptoms are a common experience among cancer patients, and they can be caused by a variety of factors such as the disease itself and side effects of cancer treatments, in addition to psychological distress. As such, further research is needed to better understand this relationship and the potential underlying mechanisms, as well as the potential of mindfulness-based interventions for alleviation of somatic symptoms in patients with cancer.

### Study limitations

While our results appear valid and robust, they should be interpreted in the context of the study design. Indeed, a single type of cancer at an early stage was included, and future work should consider studying a more diverse sample of patients with cancer. Additionally, the healthy sample was not tested at a second time point, limiting the ability to perform analyses of differential progress of MAAS scores and of symptoms, relative to the population of patients with cancer. Furthermore, the study relied solely on self-report questionnaires, with no use of alternative measures of mindfulness or psychological symptoms, which may have introduced response biases. The use of a single data source also limits the validity of the findings and highlights the need for multi-method assessment in future studies. Finally, although the MAAS was developed to assess dispositional mindfulness – confirmed here as a relatively stable trait (Brown & Ryan, 2003) – it remains unclear if this scale is suitable for detecting the effects of mindfulness-based interventions.

## Conclusions

In the present study, we assessed dispositional mindfulness in patients with early-stage BC at diagnosis, before systemic treatment, and again 12 months later, alongside measures of affective and somatic symptoms. Dispositional mindfulness was assessed using the MAAS, that we found to be valid in the study population of interest and have the expected psychometric properties of the scale as described in previous studies. Most importantly, we found that mindfulness measured by MAAS, while invariant between patients and healthy volunteers, and stable across time, predicted affective symptoms 12 months later. MAAS should thus be further tested in a clinical setting to assist in management of affective symptomatology in patients with cancer, potentially contributing towards better treatment outcomes, and ultimately promoting improvements in quality of life.
